# Pain Processing in Elite and High-Level Athletes Compared to Non-athletes

**DOI:** 10.3389/fpsyg.2020.01908

**Published:** 2020-07-28

**Authors:** Susann Dahl Pettersen, Per M. Aslaksen, Svein Arne Pettersen

**Affiliations:** ^1^Department of Psychology, UiT The Arctic University of Norway, Tromsø, Norway; ^2^School of Sport Sciences, UiT The Arctic University of Norway, Tromsø, Norway

**Keywords:** pain, athlete, experimental, fear of pain, grit, personality, endurance athletes, soccer players

## Abstract

**Background:**

Previous studies shows that elite and high-level athletes possess consistently higher pain tolerance to ischemic and cold pain stimulation compared to recreationally active. However, the data previously obtained within this field is sparse and with low consistency.

**Purpose:**

The aim of the present study was to examine the difference in pain perception between elite and high-level endurance athletes (cross country skiers and runners), elite soccer players and non-athletes, as well to explore the impact of psychological factors on pain processing.

**Methods:**

Seventy one healthy volunteers (33 females and 38 males) participated in the study. Soccer players (*n* = 17), cross country skiers (*n* = 12), and long-distance runners (*n* = 3) formed the athlete group, with 39 non-athletes as controls. Big-five personality traits, fear of pain and Grit (perseverance and passion for long-term goals) were measured prior to induction of experimental pain. Pain threshold and intensity was induced by a PC-controlled heat thermode and measured by a computerized visual analog scale. Pain tolerance was measured by the cold pressor test (CPT).

**Results:**

Elite and high-level athletes had increased pain tolerance, higher heat pain thresholds, and reported lower pain intensity to thermal stimulation. Endurance athletes (cross country skiers and long-distance runners) had better tolerance for cold pain compared to both soccer-players and non-athletes. Furthermore, endurance athletes reported lower pain intensity compared to non-athletes, whereas both endurance athletes and soccer players had higher heat pain thresholds compared to non-athletes. Fear of Pain was the only psychological trait that had an impact on all pain measures.

**Conclusion:**

The present findings suggest that sports with long durations of physically intense activity, leveling aerobic capacity, are associated with increased ability to tolerate pain and that the amount of training hours has an impact on this tolerance. However, the small sample size implies that the results from this study should be interpreted with caution.

## Introduction

Pain is an integral part of exercise and sports (O’Connor and Cook, 1999). When engaging in exercise, pain emerges as a natural consequence of intramuscular pressure, muscle distortion and a build-up of deleterious metabolites in the muscle. A general finding is that high-level athletes possess consistently higher experimental pain tolerance to ischemic and cold pain stimulation compared to normally active controls (Tesarz et al., 2012) and some of the explanations proposed are coping strategy use, ignoring pain or team culture (Deroche et al., 2011). Contrary, the findings on pain threshold and pain sensitivity (pain intensity) are mixed with some studies showing reduced thresholds in athletes compared to non-athletes, whereas other studies find results in the opposite direction (Tesarz et al., 2012; McDougall et al., 2020). The type of sport is probably a relevant cause for the divergent findings, and one study have showed that different sports are associated with different tolerance, thresholds and sensitivity for pain (Assa et al., 2019), regardless of the type of noxious stimuli.

Pain threshold is the minimum stimulus intensity that is *usually* perceived as painful, whereas pain tolerance is defined as either the length of time an individual is willing to endure a noxious stimulus, or the maximal stimulus intensity that one will endure (O’Connor and Cook, 1999). Pain sensitivity is the subjective rating of the intensity of a standardized stimulus, that typically induce nociceptive activation (Nielsen et al., 2005). Hence, athletes in contact sports with physical contact and explosive use of muscles might develop higher pain thresholds due to habituation compared to endurance sports with less acute pain caused by collisions and muscle injuries, whereas endurance sports relying on longstanding activity and cardiorespiratory fitness might increase the tolerance for pain stimulation.

High volume of aerobic training is the main concept to achieve high aerobic fitness and World-class cross-country skiers have previously reported 800–950 annual training hours (Tonnessen et al., 2014; Sandbakk et al., 2016). Football (soccer) on the other hand is an open loop sport where the players from a physiological perspective need to develop aerobic and anaerobic capacity, strength and speed requiring a different training modality compared to endurance athletes. Professional soccer players execute less training hours per year compared to cross-country skiers, around 700 (Ward et al., 2004). Thus, the differences in training regimen could cause differences in how painful stimulation is processed and tolerated between endurance athletes and soccer players.

There are several potential psychological mediators and moderators of the relationship between physical exercise and pain perception. Previous studies have suggested that general coping skills and self-efficacy could moderate how painful stimulation is perceived and tolerated (Deroche et al., 2011). However, coping-skills and self-efficacy are broad concepts not specific to pain perception and tolerance and might therefore be less suited as predictors for pain perception compared to traits directly measuring emotional reactions toward pain. Fear of pain is a dispositional trait directly associated with pain perception that have been found to be predictive for experimental pain report in numerous studies (McNeil and Rainwater, 1998), and it is reasonable to expect that Fear of pain could serve as a predictor for pain reports in athletes.

In sports, most personality research has revolved around the Big-Five personality traits and or/hardiness, resilience, mental toughness, and coping (Sarkar and Fletcher, 2014; Meyer et al., 2017). Another character trait, which has been used in studies regarding motivational aspects of sports, is Grit. Grit is a personality trait defined as passion and perseverance for long-term goal achievement (Duckworth et al., 2007) that might add more insight to differences in pain tolerance. Grit has been linked to conscientiousness (Rimfeld et al., 2016), and high levels of conscientiousness has been associated with lower levels of experienced pain, but there is little to no research regarding how scores on the Grit-scale correlate with pain perception in athletes.

In the present study, the main aims were to test whether common experimental pain measures of tolerance, intensity, and pain thresholds differ between endurance athletes and soccer players, and whether relevant psychological traits could explain differences between pain processing in athletes and non-athletes. By further investigating athletes’ pain perception, we hoped to bring more clarity to the field, which previously have shown contradictory findings. Identifying underlying mechanisms which modify tolerance and thresholds of pain, may also contribute to developing effective methods of exercise for the relief of symptoms in pain patients.

We tested the following hypotheses: Endurance athletes should display higher tolerance for cold pain, soccer players should have higher pain thresholds, and both athlete groups should report lower pain intensity compared to non-athletes. Furthermore, we expected that increased FPQ and Grit should be negatively associated with pain tolerance and thresholds, and positively associated with pain intensity reports regardless of groups.

## Materials and Methods

### Participants

The sample consisted of 72 healthy participants. Data from one participant (non-athlete) was removed from the analyses, due to reporting of weekly training hours more than 3 SD from the mean of the non-athlete group. Hence, 71 participants were included in the analyses.

The participants had a mean age of 23.79 years (range 18–37, *SD* = 4.43), 47.14% of whom were women. Athlete participants were comprised of soccer players (*n* = 17), aerobic endurance athletes (*n* = 15), 45.16% of whom were women. The athletes were recruited from several sport clubs, all competing at the highest national level in Norway. The recruitment of the athletes was conducted by contacting the coaching staff in each individual soccer, ski and long-distance running team by mail and through a poster located at the Alfheim Research and Exercise lab. The email consisted of an inquiry for participation and information regarding the procedure of the project. The control group was recruited via a poster located at the university area. Information regarding the study was provided both orally and in writing. All participants signed a written informed consent. The consent form emphasized that participants could not partake in the study if they were pregnant, had a history of ongoing disease or previous serious diseases such as heart conditions (including increased blood pressure), metabolic disorders, mental disorders, damaged skin on the forearms, neurological illnesses or brain damage, or damage in the central nervous system. Volunteers who used any type of prescribed medications could not participate, except for birth control pills and asthma medicine. The subjects included in the study received a gift card with a value of 200 NOK (about 22 USD/20 EUR) for participating. The experimental protocol was conducted in accordance with the Helsinki Declaration and was approved by the Regional Committee for Medical Research in North Norway (REK).

### Experimenters

Fifty-two of the participants were tested by a female studying clinical psychology at the University of Tromsø. The remaining subjects were tested by a male professor employed at the same university. Interaction between experimenter and subject was standardized in a written procedure.

#### Pain Apparatus

A cold pressor pain task was accomplished using a computer-controlled water circulator (JeioTech, South Korea). The temperature was maintained at 2°C.

Heat-pain was induced by contact heat stimulation (30 × 30 mm aluminum contact thermode, Pathway; Medoc, Israel) attached to the surface of the left volar forearm.

Pain intensity was measured continuously during stimulation by a 0–100 Computerized Visual Analog Scale (COVAS, Medoc, Israel) where 0 equaled no pain sensation and 100 equaled the most intense pain sensation imaginable. Blood pressure and heart rate were measured with a standard electronic blood pressure device (Microlife, Widnau, Switzerland). Systolic/diabolic blood pressure and heart rate were registered before and after the cold pressor test (CPT).

### Procedure

The experiment took place at Alfheim Research and Exercise lab, and at the Department of Psychology, UiT The Arctic University of Norway. The room temperature was kept stable at 21 degrees at both sites, and both labs were shielded from external sounds. Participants were tested individually. Upon arrival the participants received information about the experiment and signed the consent form. They were told that the purpose of the study was to examine how physical exercise and personality affect the experience of physical pain. They filled out the BFI-10, FPQ-III, and Grit-S scale, before being placed in a comfortable chair. Blood pressure and heart rate a were measured seated before executing the experiment.

Thereafter, a CPT and a MEDOC Pathway somatosensory stimulator apparatus to conduct quantitative sensory testing (QST) was applied. Participants were instructed to submerge their right hand up to and including the wrist into cold water (2°C). Subjects were told to keep their hand in the water as long as possible and that they could remove their hand at their discretion, but after 3 min they were instructed to remove it and the test was terminated. Upon finishing the test, blood pressure, heart rate and the hand skin-surface temperature measurement was repeated.

Heat-pain threshold (HPth) was measured using a MEDOC Pathway somatosensory stimulator. We used the “method of limits,” where the pain intensity level is set below pain threshold and subsequently increased until the stimuli is perceived as painful. The thermode was attached to the left volar forearm for all participants, except two subjects who had tattoos on the left arm and therefore undertook heat pain testing on the right volar forearm. The thermode had a baseline temperature of +32°C when applied to the skin. The temperature increased by 1°C per second and an upper safety limit was set at 52°C. Participants were instructed to press a button when the sensation changed from warmth to pain. Upon clicking the button, temperature was registered, and the temperature returned to baseline with a fall rate of 8°C/s. The measurement was repeated five times and HPth was calculated as the mean of the five measurements. Pain threshold measurements and analyses were performed according to recommendations for QST for pain studies (Rolke et al., 2006).

Participants subsequently went through a pain intensity test, as a measure of pain sensitivity. Subjects were asked to rate their pain continuously on the computerized visual analog scale. The test started at baseline temperature (32°C), increased by 10°C/s and kept a stable temperature of 47,5°C for 30 s, before returning to baseline. The target temperature was selected based on previous studies in our lab showing that a tonic heat stimuli >47°C is rated as painful in most healthy participants (Aslaksen et al., 2014, 2016). Upon finishing the two intensity tests, the thermode was moved 2 cm from the original spot toward the elbow, to avoid hyperalgesia. The pain threshold and pain intensity test were then repeated. The total duration of the experimental procedure was approximately 25 min for each participant.

### Instruments

Grit is a personal quality defined as perseverance and passion for long-term goals (Duckworth et al., 2007). Grit was measured by an eight item self-report questionnaire (Grit-S). The items were rated on a five-point Likert scale (1 = not at all like me, 5 = very much like me). Development and validation of the Grit-S scale showed acceptable internal consistency, with alphas ranging from 0.73 to 0.83 (total score) across four samples (Duckworth and Quinn, 2009).

BFI-10 is a short scale version of the Big Five Inventory, which consists of 44 items (Rammstedt and John, 2007). The 10-item self-report measure containing short items assessing the Big Five factors (Openness, Conscientiousness, Extraversion, Agreeableness, and Neuroticism). Items were answered using a five-point Likert scale (1 = strongly disagree, 5 = strongly agree). BFI-10 has shown acceptable internal consistency (extraversion – α = 0.45, agreeableness – α = 0.24, conscientiousness – α = 0.62, neuroticism – α = 0.55, and openness – α = 0.36) (Balgiu, 2018).

FPQ-III (Fear of Pain Questionnaire-III) (McNeil and Rainwater, 1998) contains 30 items consisting short phrases depicting painful situations. The items were rated on a five-point Likert scale (1 = no pain, 5 = severe pain). The questionnaire consists of three 10-item subscales: fear of severe pain (e.g., “Breaking your arm”), medical pain (e.g., “Having a blood sample drawn with a hypodermic needle”), and minor pain (e.g., “Getting a paper-cut in your finger”). Respondents were instructed to rate the degree of anticipated pain related to each item. It is established that the questionnaire has a good internal consistency (total score, α = 0.92; severe pain, α = 0.88; minor pain, α = 0.87; and medical pain, α = 0.92) and good test–retest reliability (total scale, α = 0.74; severe pain, α = 0.69; minor pain, α = 0.73; and medical pain, α = 0.76) (McNeil and Rainwater, 1998). The Norwegian version of the FPQ was used in the present study (Vambheim et al., 2017).

### Statistical Analysis

The statistical analysis was performed with SPSS version 26.0 (SPSS, Inc., Chicago, IL, United States). The distribution of data for the pain tolerance test (CPT) was significantly deviant from a normal distribution shown by the Shapiro–Wilk test (*p* < 0.001) and by visual inspection of Q-Q- and box plots. Data distributions for pain intensity and pain thresholds were not significantly different from normal distributions shown by the Shapiro–Wilk test (both *p*’s > 0.13). Correlational analyses were performed with Spearman correlations for data with a non-normal distribution, and Pearson correlations were used for data with normal distributions. Cox regressions were used to test group differences and the effect of covariates on pain tolerance (time in the CPT). Withdrawal of the hand before the 180 s limit was coded as 1 (event) and if the participant sustained the CPT for 180 s the participant was censored (coded 0). Linear mixed models (LMM) were used to analyze the repeated data for pain intensity and pain thresholds. LMM’s were chosen because these analyses are suitable for analyzing data with unequal group sizes, handle missing data without losing power in the analyses compared with standard general linear models, and allows for combinations of both fixed and random effects (Aslaksen et al., 2018).

Model fits were assessed with the −2 restricted log likelihood criteria. For all reported LMM analyses, a diagonal covariance matrix was superior to autoregressive matrices and a compound symmetry matrix, shown by lower −2 restricted log likelihood values. The repeated factor had two levels, trial 1 and trial 2. The participants were assumed to induce individual variance in addition to the variance associated with their group belonging, and the participants individual variance was the only random factor in the LMMs. Bonferroni corrections were used to correct *p*-values for the family wise error rate when comparing levels within the group variable in the LMM analyses. Cohens *d* were calculated for estimation of effect sizes for significant differences between the groups in the LMM analyses. *P*-values < 0.05 were considered significant.

## Results

The sample consisted of 38 men and 33 women, with a mean age of 23.79 years. The sample consisted of 32 athletes divided into three groups of soccer players (*n* = 17), cross-country skiers (*n* = 12), and long-distance runners (*n* = 3). The cross-country skiers and the long-distance runners were established as an endurance athlete group in analysis comparing in-group effects. The non-athlete group spent an average of 3.92 h per week exercising, whilst the elite athlete group spent an average of 16.5 h per week. There were no significant gender differences in the pain data (tolerance, intensity, thresholds), all *p*’s > 0.09. See Table 1 for means and group-comparisons.

**TABLE 1:** Statistics, Mean values and standard deviations (SD): One-way ANOVA.

Measures	Endurance athletes (*SD*)	Soccer-players (SD)	Non-athletes (*SD*)	*F*-value	*p-*value	*Post-hoc*
N	15	17	39			
Weekly exercise (hours)	17.53 (2.26)	15.59 (2.85)	3.92 (2.74)	174.18	<0.001	E & S > N
Age (years)	21.67 (3.62)	24.47 (5.20)	24.31 (4.21)	2.27	0.11	
Grit	3.73 (0.20)	3.39 (0.26)	3.30 (0.34)	11.21	<0.001	E > S & N
FPQ Minor pain	15.47 (5.03)	18.82 (6.35)	19.82 (6.06)	9.22	0.061	
FPQ Severe pain	33.27 (8.07)	34.35 (5.75)	34.21 (7.12)	0.12	0.89	
FPQ Medical pain	19.67 (5.86)	23.94 (7.59)	23.31 (6.98)	1.87	0.16	
FPQ total	68.40 (15.30)	77.12 (16.73)	77.33 (17.77)	1.60	0.21	
BFI-10 Extroversion	3.40 (1.11)	3.82 (0.88)	3.63 (0.98)	0.74	0.48	
BFI-10 Agreeableness	4.20 (0.37)	3.53 (0.45)	3.80 (0.76)	4.57	0.01	E > S
BFI-10 Conscientiousness	4.60 (0.54)	3.82 (0.58)	3.52 (0.97)	9.44	<0.001	E > S & N
BFI-10 Neuroticism	2.37 (0.95)	2.06 (0.68)	2.51 (0.85)	1.73	0.19	
BFI-10 Openness	2.87 (1.11)	3.21 (1.00)	3.55 (0.88)	2.77	0.07	
CPT* (time in seconds)	179.67 (0.90)	113.90 (71.54)	116.78 (64.38)	6.85	0.002	E > S & N
Pain threshold 1** (°C)	47.62 (1.05)	47.88 (2.16)	46.56 (1.97)	3.69	0.03	–
Pain intensity 1**	45.53 (12.79)	51.88 (25.30)	59.38 (29.07)	1.71	0.19	
Pain threshold 2 ** (°C)	48.26 (0.97)	48.15 (1.28)	46.89 (1.38)	9.11	<0.001	E & S > N
Pain intensity 2 **	37.93 (17.73)	45.44 (23.89)	53.72 (29.76)	2.07	0.134	

*Post-hoc* tests with Bonferroni corrections. E, Endurance athletes; S, Soccer players; N, Non-athletes. Grit: Measured on a five-points Likert scale. FPQ, Fear of Pain Questionnaire, measured on five-points Likert scales. BFI-10: Big Five Inventory-10, measured on five-points Likert scales. CPT: Cold Pressor Test; Pain threshold: Mean value of five measurements; Pain intensity: Highest value on visual analog scale (0–100). *Mann–Whitney *U*-Test with Bonferroni corrections applied. **Soccer players *n* = 16.

### Pain Tolerance

The association between pain tolerance for the whole sample measured by time in the CPT, personality traits, fear of pain, and grit were analyzed by Spearman correlations. Tolerance for the CPT was significantly positively associated with the personality trait conscientiousness (*r* = 0.38, *p* = 0.001), the grit total score (*r* = 0.46, *p* < 0.001), and negatively associated with the total score for fear of pain (*r* = −0.51, *p* < 0.001).

**FIGURE 1:**
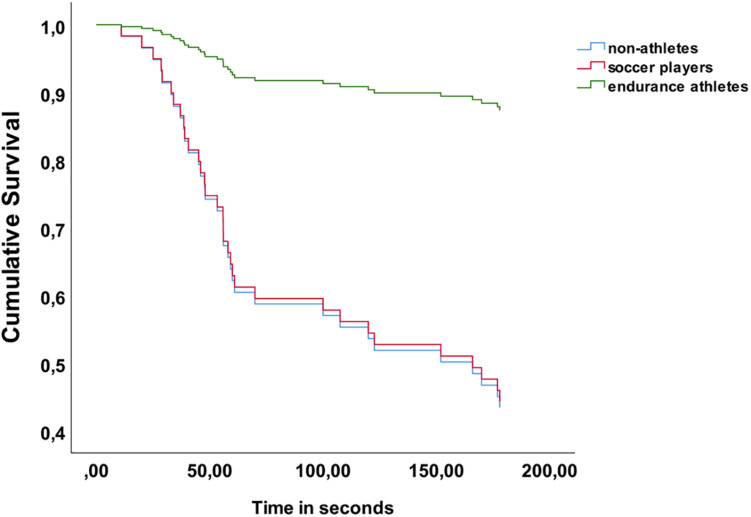
Cox-regression model for pain tolerance.

Cox regression analysis was employed to test the impact of the covariates significantly associated with CPT data on the tolerance time for the three sub-groups. 38 (53.5%) participants tolerated the CPT for the maximum allowed time of 180 s, whereas 33 (46.5%) discontinued before the time limit. The Cox survival analyses was first tested with group (soccer players, endurance athletes, non-athletes) as the only covariate, then secondly as a model with group as categorical covariate, and FPQ, grit, training volume and the personality trait conscientiousness as continuous covariates. The model with only the categorical covariate (group) showed that there was a significant difference between the groups where endurance athletes had higher probability for tolerance of the CPT until the maximum allowed time of 180 s compared to both soccer players (*B* = 1.8, Wald = 5.26, OR = 6.02, *p* = 0.02) and non-athletes (*B* = 1.82, Wald = 6.05, OR = 6.17, *p* = 0.014). When entering the significant covariates from the correlation analysis (grit, FPQ, and conscientiousness) together with group and training volume per week, the significant differences between the groups changed. When adjusting for the aforementioned covariates the non-athlete group showed a significantly higher probability for tolerating the CPT compared to soccer players (*B* = 2.32, Wald = 10.08, OR = 10.19, *p* < 0.001) whereas there were no significant differences between non-athletes and endurance athletes, and endurance athletes and soccer players. Increased training volume increased the likelihood of tolerating the total CPT time (*B* = −0.18, Wald = 4.60, OR = 0.84, *p* = 0.032). Increased Fear of Pain was associated with lower pain tolerance (*B* = 0.04, Wald = 17.57, OR = 1.03, *p* < 0.001). Increased level of grit had a non-significant tendency to be associated with pain tolerance (*B* = −1.47, Wald = 3.42, OR = 0.23, *p* = 0.06), where higher grit was associated with reduced risk of discontinuing the CPT. The personality trait conscientiousness had no impact (*B* = 0.18, Wald = 0.77, OR = 1.2, *p* = 0.38) on pain tolerance in this model. See Figure 1 for an overview of the Cox model.

### Pain Intensity and Heat Pain Thresholds

The association between pain intensity, pain thresholds, personality traits, FPQ and grit were tested with Pearson correlations. Both pain measures showed significant correlations with FPQ (pain intensity: *r* = 0.38, *p* < 0.001, pain threshold: *r* = −0.28, *p* = 0.001), and Grit. None of the other psychological measures reached significance. Thus, FPQ was included as a covariate in the LMM with pain intensity and pain threshold as dependent variables.

Firstly, models for pain intensity and pain thresholds including group and trial (repeated effect) were fitted. For pain intensity, there was a main effect of group [*F*(2,136.85) = 3.83, *p* = 0.024] with higher pain intensity reports in the non-athlete group compared to the endurance athlete group (*p* = 0.027, *d* = 0.31), with no other significant group differences. In the pain threshold data, the main effect of group was significant [*F*(2,121.33) = 12.80, *p* < 0.001], where non-athletes had significantly lower pain thresholds compared to both soccer players (*p* < 0.001, *d* = 0.46) and endurance athletes (*p* = 0.002, *d* = 0.42).

When including the covariates from the correlation analyses together with training volume in the model, there were no significant differences between the groups on pain intensity, and the only significant variable was the total Fear of Pain score (*B* = 0.50, SE = 0.13, *t* = 3.92, *p* < 0.001), where higher FPQ was associated with increased pain intensity reports. The LMM for pain thresholds showed that training volume (*B* = 0.15, SE = 0.04, *t* = 3.35, *p* = 0.001) and FPQ (*B* = −0.02, SE = 0.01, *t* = −2.22, *p* = 0.028) had main effects on heat pain thresholds, where increased number of training hours per week and lower FPQ scores were associated with higher thresholds. See Table 2 for the model of fixed effects including covariates. The random effect of individual variance was significant in both the analyses of pain intensity and pain threshold, both Wald Z were >5.39, and both *p*’s < 0.001. Thus, the effect of individual differences was significant for both models.

**TABLE 2:** Linear mixed models for pain intensity and pain thresholds.

Variable:	Df Numerator/Denominator	*F*	*p*
**Heat pain intensity: Model 1**			
Group	2/136.85	3.83	0.024
Trial	1/136.76	2.07	0.15
**Heat pain intensity: Model 2**			
Group	2/116.68	0.01	0.99
Trial	1/120.09	2.28	0.13
Weekly exercise	1/116.62	0.82	0.37
FPQ	1/116.80	15.36	<0.001
Grit	1/116.94	0.70	0.41
**Heat pain thresholds: Model 1**			
Group	2/121.33	12.80	<0.001
Trial	1/122.91	1.98	0.16
**Heat pain thresholds: Model 2**			
Group	2/116.68	1.08	0.34
Trial	1/120.09	2.24	0.14
Weekly exercise	1/116.68	11.20	0.001
FPQ	1/116.80	4.92	0.028
Grit	1/116.94	0.02	0.90

*FPQ, Fear of Pain. Trial consisted of test 1 and test 2.*

### Blood Pressure and Heart Rate

Pearson correlations showed that there were no significant associations between systolic- and diastolic blood pressure, heart rate per minute and the included pain measures. All *p*’s > 0.05.

## Discussion

The aims of this study were to test whether common experimental pain measures of tolerance, intensity and pain thresholds differed between endurance athletes and soccer players, and whether relevant psychological traits could explain differences between pain processing in athletes and non-athletes. The main findings showed that elite and high-level athletes had increased pain tolerance, higher heat pain thresholds, and reported lower pain intensity to thermal stimulation. However, the type of sport did matter. Endurance athletes had increased tolerance for cold pain compared to both soccer-players and non-athletes. Furthermore, endurance athletes reported lower pain intensity compared to non-athletes, whereas both endurance athletes and soccer players had higher heat pain thresholds compared to non-athletes.

Increased pain tolerance in endurance athletes was in accordance with our hypothesis and suggest that sports with long duration of physically intense activity are associated with increased ability to tolerate pain. Our results are in line with findings in a meta-analysis (Tesarz et al., 2012) and more recent studies showing that pain tolerance is generally higher in athletes compared to non-athletes. Furthermore, training volume per week increased the likelihood of sustain the CPT to the maximum allowed time of 180 s. The amount of training hours was not significantly different between endurance athletes and soccer players, but these results suggest that aerobic endurance training increases the probability of tolerating cold pain stimulation compared to mixed aerobic-anaerobic activity typical for soccer training.

Nonetheless, the included measures of Grit and Fear of Pain (FPQ) affected the probability for tolerating the CPT showing that psychological traits have a significant impact on pain tolerance regardless of training volume and type of sport. The group of endurance athletes had higher Grit-scores compared to both soccer players and non-athletes, and higher Grit scores were associated with increased time in the CPT. To our knowledge, this is the first study to assess the association between Grit and experimental pain in a sample of athletes. However, a recent meta-analysis has provided a question of the merit of grit construct in performance realms, suggesting a need to investigate Grit theory and measurement validity in sport before assuming construct relevance in athlete samples (Credé et al., 2017). Another possible explanation of this finding is the inherent difference between sports achievement that mostly is a voluntary compared to academic achievement which is more an obligatory endeavor (Meyer et al., 2017). Meyer et al. (2017) recommend avoiding terms like grit in sports, because little to any evidence supports the existence of the construct or its significance in the sports domain. Increased fear of pain reduced the ability to tolerate the CPT. Several previous studies in samples stated as non-athletes have shown the same findings as the present study, where increased fear of pain was negatively associated with the ability to endure pain (Vambheim et al., 2017; Patanwala et al., 2019). Thus, FPQ might be a trait that independent of training status affect how the individual perceive painful experiences and thereby influences the behavior when painful stimulation is present. The influence of FPQ on pain was also shown in a sample of triathletes (Geva and Defrin, 2013), where higher FPQ was associated with better pain modulation compared to non-athletes. However, it should be noted that the sample size in the present study was small, and the results from the extended statistical models with several covariates should be interpreted with caution.

Pain thresholds were higher in both athlete groups compared to non-athletes, and several previous studies (Guieu et al., 1992; Granges and Littlejohn, 1993; Raudenbush et al., 2012; Flood et al., 2017) have shown similar findings. However, there were no difference between soccer players and endurance athletes on this measure. The difference in training between the endurance athletes included in our study (cross country skiers and runners) and the soccer players were probably not enough to produce differences in pain thresholds. A recent study (Assa et al., 2019) that included more distinct types of sports, showed that athletes in strength sports, e.g., weightlifters, hammer- and shotput throwers had increased heat pain thresholds compared to endurance athletes and controls. Like the pain tolerance measure, FPQ had also a significant impact on pain thresholds together with training volume per week. Training volume has previously been associated with increased pressure pain thresholds (Kuppens et al., 2019), but no previous study has tested whether the association between training hours per week and heat pain thresholds. In the pain intensity data, the non-athletes reported higher pain levels compared to the elite athletes, whereas the difference between soccer players and endurance athletes was non-significant. There are few studies that directly have assessed pain intensity in athletes vs. non-athletes, but studies testing conditioned pain modulation (CPM) suggests that this process is more efficient in athletes vs. non-athletes (Tesarz et al., 2013; Flood et al., 2017) lending support to the finding in the present study. On the other hand, a recent meta-analysis suggests that the tendency to better CPM in athletes is highly variable across studies, possibly due to differences in training volume across sport types (McDougall et al., 2020). The only psychological trait measure that had an impact on pain intensity was fear of pain measured by the FPQ questionnaire. Similar to the other pain measures, the finding that FPQ affects pain intensity is shown in numerous studies outside studies of sports (Lyby et al., 2011; Aslaksen and Lyby, 2015; Corsi and Colloca, 2017; Vambheim et al., 2017). However, in the present data there was no difference between the groups on the FPQ, and the FPQ measure seems to affect pain independently of athletic status. Personality traits measured by the BFI-10 had limited impact on the included measures of pain, and the only significant association was the correlation between pain tolerance and conscientiousness. However, this association was abolished when including other psychological factors, suggesting that traits which directly taps into pain are more predictive for behavioral pain responses compared to higher-order personality traits (Lee et al., 2010).

The main findings in the present study were constituted of generally better ability to tolerate and sustain painful experimental stimulation in elite athletes compared to non-athletes. The design in the present study cannot provide causal explanations for why these findings emerge, either in the present study or previous similar studies. A possible explanation for why elite athletes report lower pain compared to non-athletes is the repetitive exposure for low intensity pain which might induce physical and mental tolerance for pain. A recent study showed that elite athletes had reduced neural responses to anticipation of low-intensity pain stimulation compared to non-athletes, suggesting that the previous repetitive experience of low-intensity pain alter central pain processing (Geisler et al., 2019). Hence, future studies should include larger samples and involve measures of cerebral pain processing in order to provide more information on how physical training make an effect on cerebral pain processing. Fear of Pain was the only psychological trait that had an impact on all pain measures and the level of FPQ was not significantly different between groups. As stated above, this is in line with our expectations but does also suggest that psychological factors associated with pain might be highly influential in top athletes, and even affect their performance. For non-athletes or recreational athletes, the tendency to be fearful of pain might reduce their activity levels (Tripp et al., 2007) and thereby increase the probability of negative consequences of inactivity (Picavet and Schuit, 2003).

Finally, the present study faces the challenge of ecological validity. Even though consensual pain induction techniques can be administered in a controlled and safe environment, individuals know that the induced pain can be terminated at any time. This does not replicate the same nature of the pain experienced in training or competition by the athletes. The same challenge applies for the type of pain induced, in this case heat and cold pain. The soccer players experience periods of pain associated with short bouts of supramaximal intensity and receiving blows from opponents or the ball (Mohr et al., 2003). The endurance athletes experience a prolonged interoceptive pain caused by persistent intense activity close to maximal oxygen uptake (Sagelv et al., 2018). In addition, the pain athletes’ experience is often in situations with elevated levels of adrenaline. The challenge for future research is to find useful procedures for distinguishing what mechanisms underly pain perception, in a real-world sport situation, and thereby develop strategies for “pushing through the pain” not caused by injury. It would be advisable to run such studies with a larger sample size.

## Data Availability Statement

The raw data supporting the conclusions of this article will be made available by the authors, without undue reservation.

## Ethics Statement

The studies involving human participants were reviewed and approved by the Regional Committee for Medical Research in North Norway (REK). The patients/participants provided their written informed consent to participate in this study.

## Author Contributions

SDP designed the experiment, collected and analyzed the data, and wrote the manuscript. PA designed the experiment, obtained funding, collected and analyzed the data, and wrote the manuscript. SAP designed the experiment, obtained funding, and wrote the manuscript. All authors contributed to the article and approved the submitted version.

## Conflict of Interest

The authors declare that the research was conducted in the absence of any commercial or financial relationships that could be construed as a potential conflict of interest.
